# Atherosclerosis Is an Inflammatory Disease which Lacks a Common Anti-inflammatory Therapy: How Human Genetics Can Help to This Issue. A Narrative Review

**DOI:** 10.3389/fphar.2018.00055

**Published:** 2018-02-06

**Authors:** Cristiano Fava, Martina Montagnana

**Affiliations:** ^1^General Medicine and Hypertension Unit, Department of Medicine, University of Verona, Verona, Italy; ^2^Clinical Biochemistry Section, Department of Neurosciences, Biomedicine and Movement Sciences, University of Verona, Verona, Italy

**Keywords:** atherosclerosis, inflammation, genetics, anti-inflammatory drugs, genome wide association study, DNA sequencing

## Abstract

Atherosclerosis is a multifactorial disease triggered and sustained by different risk factors such as dyslipidemia, arterial hypertension, diabetes mellitus, smoke, etc. Since a couple of decades, a pivotal role for inflammation in its pathogenesis has been recognized and proved at molecular levels, and already described in many animal models. Despite all this knowledge, due to the complexity of the specific inflammatory process subtending atherosclerosis and to the fact that inflammation is also a protective response against microorganisms, no anti-inflammatory therapy has been rendered available in the therapeutic armamentarium against atherosclerosis and vascular events till 2017 when canakinumab in the first *ad-hoc* randomized clinical trial (RCT) proved for the first time that targeting specifically inflammation lowers cardiovascular (CV) events. From the genetic side, in the 90's and early 2000, several genetic markers in inflammatory pathway have been explored searching for an association with athero-thrombosis which gave seldom consistent results. Then, in the genomic era, plenty of genetic markers covering most of the genome have been analyzed at once without *a priori* information. The results coming from genome wide association studies (GWAS) have pinpointed some loci closed to inflammatory molecules consistently associated with atherosclerosis and CV consequences revamping the strict link between inflammation and atherosclerosis and suggesting some tailored target therapy. Whole-exome and whole-genome sequencing will come soon showing new and old loci associated with atherosclerosis suggesting new molecular targets or underlying which inflammatory pathway could be most attractive to target for blocking atherosclerosis even in its early stages.

## Introduction

Atherosclerosis is an inflammatory disease (Ross, 1999; Libby, 2012). Accumulation of leukocytes in the subendothelial space is an early step in the formation of atherosclerotic lesions. Then, other inflammatory cells follow and participate in all the processes starting from the “fatty streak” and leading to advanced atherosclerotic lesions triggering clinical events. In particular a pivotal role has been recognized for monocyte-derived macrophages, which become “foam cells,” dendritic cells, lymphocytes (both T and B), and mast cells. Lots of adhesion molecules or receptors for leukocytes expressed on the surface of the arterial endothelial cell probably participate in the recruitment of leukocytes to the nascent atheroma. Proinflammatory cytokines can act at different stages in the process: interleukin 1 (IL-1) and tumor necrosis factor (TNF) can regulate the expression of adhesion molecules involved in early and late leukocyte recruitment. Indeed, IL-1 and TNF can induce local production of growth factors, including fibroblast growth factors (FGF) and platelet-derived growth factor (PDGF) which attract smooth-muscle cells from the tunica media into the intima. Finally, other cytokines and growth factors may be important in the evolution to a more advanced fibrous plaque which may be protective against plaque rupture: i.e., the transforming growth factor β (TGF-β) stimulates whereas interferon γ (IFN-γ) counteracts interstitial collagen production by smooth-muscle cells (Ross, 1999; Libby, 2012).

C- reactive protein (CRP) is an acute phase reaction protein, produced by the liver and triggered especially by interleukin-6 (IL-6) which is often used in the clinic as an inflammatory marker in “classical” inflammatory disease such as infections, vasculitis, tumors. When all these conditions are excluded, CRP, even in the lower range of detection, could be useful to monitor subclinical inflammatory state deriving from atherosclerosis (Libby and Ridker, 2004).

The Jupiter trial have already shown that patients with 2.0 mg/L or higher level of CRP benefit from statin therapy to lower their CV risk. But of course in that trial not only CRP (by 37%) but especially LDL cholesterol decreased (nearly by 50%) along with all the end-points: myocardial infarction (MI), stroke, arterial revascularization, hospitalization for unstable angina, or death from CV causes (Ridker et al., 2008). Thus, after this trial it was still plausible that the reduced LDL-cholesterol levels and not the reduced grade of inflammation mostly contributed to this beneficial result.

Secretory phospholipase A2 (sPLA2) and lipoprotein-associated phospholipase A2 (Lp-PLA2) were identified in animal and observational studies in humans, as potential risk factors for coronary heart disease due to their putative effects on lipids and inflammation (Rosenson et al., 2010; Wang et al., 2011). Nevertheless, two randomized clinical trials (RCTs), exploring the possible efficacy of darapladib, a Lp-PLA2 inhibitor, in patients after an acute coronary syndrome (ACS) or stable coronary atherosclerosis, did not find any difference in CV and cerebrovascular events as compared to placebo (O'Donoghue et al., 2014; STABILITY Investigators et al., 2014). A RCT, testing varespladib, a sPLA2 inhibitor, failed to demonstrate a beneficial CV effect and showed instead a possible increase in coronary events (Nicholls et al., 2014). A genetic variant, rs11573156 of the *sPLA2* gene was associated with lower level of sPLA2 but not with major vascular events. Thus, also Mendelian randomization analysis fails to indicate sPLA2 as a possible target for preventing CV diseases (Holmes et al., 2013).

## Targeting inflammation with old drugs

Beside the current large availability of anti-inflammatory drugs in the medical field, targeting specifically inflammation in humans remains challenging. In fact, most of available anti-inflammatory medications have adverse effects which render their use, as a chronic therapy to prevent CV events, not feasible and some of them have proved to be deleterious.

Nevertheless, at least a clue can be drawn by exploring clinical trials (which in *post-hoc* analyses evaluated these drugs for their potential role in CV risk) or observational surveys related to patients which need these drugs for other indications, such as chronic inflammatory or degenerative diseases.

Among the anti-inflammatory therapies, the non-steroidal anti-inflammatory drugs (NSAIDs) are the most common used drugs worldwide in acute inflammatory disease, chronic therapy for osteoarthritis or other painful debilitating diseases. NSAIDs effects have been explored in many trials and their potential CV effect evaluated many times. Quite recently, in a network meta-analysis including 31 trials which analyzed either NSAIDs and coxibs, both rofecoxib and lumiracoxib were associated with an increased risk of MI whereas ibuprofen and diclofenac with the risk of stroke. (Trelle et al., 2011) Two years later a comprehensive meta-analysis collected 280 trials where different anti-inflammatory agents were tested vs. placebo and 474 trials where the comparison was between anti-inflammatory agents and another NSAIDs (including coxibs) (Coxib and traditional NSAID Trialists' (CNT) Collaboration et al., 2013). Major vascular events, and especially coronary events were increased by coxibs, diclofenac, and ibuprofen. Only high-dose naproxen was associated with less vascular risk than other NSAIDs (Coxib and traditional NSAID Trialists' (CNT) Collaboration et al., 2013).

Methotrexate (MTX) is an anti-inflammatory drug widely used for the treatment of chronic inflammatory disorders such as rheumatoid arthritis and psoriasis. A systematic review and meta-analysis exploring the effect of MTX on major CV outcomes searched for cohorts, case-control studies, and randomized trials (Micha et al., 2011). In many observational studies MTX was associated with lower risk for CVD (21% reduction) and MI (18% reduction; Micha et al., 2011). The authors suggested that MTX could be a useful drug to decrease CV risk and these findings were in line with other meta-analyses (Roubille et al., 2015).

About possible effects of corticosteroids, Roubille and co-authors explored studies in patients with rheumatoid and psoriatic arthritis. Corticosteroids were associated with an increased risk of cardiovascular events regardless of the inflammatory disease (Roubille et al., 2015). This was probably due to the well-known adverse cardiometabolic effects of this class of drugs. The same meta-analysis indicated that in rheumatoid arthritis, TNF inhibitors can reduce the risk of CV events. These data confirmed findings of previous meta-analyses and large registries (Barnabe et al., 2011; Westlake et al., 2011; Low et al., 2017) but other studies did not find any significant difference (Ryan et al., 2011; Ljung et al., 2012).

Thus, TNF may represent an anti-inflammatory drug for atherosclerosis, even though its potent adverse effects and high cost makes its use for this treatment unlikely.

Another biological therapy for chronic plaque psoriasis targets interleukin 12 (IL-12) and interleukin 23 (IL-23). Two meta-analyses explored their possible effect on CV risks. The conclusion of the first meta-analysis is that anti IL-12/23 therapy is not statistically different from placebo regarding CV events, but it is underlined that 10 major adverse CV events were registered in the intervention arm as compared to 0 in the placebo group. (Ryan et al., 2011) A successive meta-analysis, including some more trials, concluded that IL-12/23 therapy increases the risk of a CV outcome (Tzellos et al., 2013).

In a phase II RCT 182 patients with NSTE-ACS, recruited in the first 48 h from onset of chest pain, were allocated to either placebo or subcutaneous IL-1 receptor antagonist (IL-1ra) for 14 days. The IL-1ra group, significantly reduced hs-CRP and IL-6 levels (*P* = 0.02). The study was underpowered to detect a difference in CV endpoints. Nevertheless, MACE despite being similar till the 3rd month were higher in the IL-1ra group at 1 year (Morton et al., 2015).

## The CANTOS study and the proof of concept

In September 2017, the results of the Canakinumab Anti-inflammatory Thrombosis Outcome Study (CANTOS) trial irrupted in the scientific field unequivocally proving for the first time that targeting specifically inflammation is useful for patients with atherosclerosis (Ridker et al., 2017).

More than 10,000 patients with a previous history of MI and levels of hs-CRP >2 mg/L were randomly assigned to three different dosages of canakinumab, an antibody which binds and blocks selectively the IL-1beta receptor (no effect observed for IL-1alpha). A control placebo group was included in the study. Due to financial considerations, the final sample size was reduced but the follow-up was extended. Interestingly the primary endpoint was reached in significantly less patients assigned to canakinumab then placebo. On the other hand, more deaths from infections were detected in the canakinumab group (Ridker et al., 2017).

Thus, a definitive point for the inflammatory hypothesis has been finally reached: atherosclerosis is an inflammatory disease which can be targeted by a specific anti-inflammatory therapy. But the drug to use remains still undefined. In fact, at least for canakinumab, there are simple considerations which will limit its widespread use: first the weight of the increase of deaths from infections may counterbalance the decrease in CV events; second the price of this therapy is still not easily affordable (Harrington, 2017).

Clinical trials to test a cheaper anti-inflammatory drug for atherosclerosis, the MTX, are still ongoing (Everett et al., 2013; Moreira et al., 2013).

Anti-inflammatory targets are probably multiplex and many drugs could target different steps in the inflammatory process. In this regard, many genetic studies and especially genome wide association studies (GWAS) have pinpointed new molecular candidates that might represent specific targets for the inflammatory process in atherosclerosis rather than other inflammatory disease. Thus, genetics could be one of the key to detect molecular targets which specifically address CV inflammation. By searching in the Pubmed database, on the 15^th^ of November 2017 and using as key words: “genome wide association study” OR “coronary” AND “atherosclerosis,” 2,858 citations were retrieved. In the remaining of the review, we focus our attention on some interesting examples between the screened studies where inflammatory loci were identified by the genome wide strategy to support the inflammatory hypothesis. Even if we acknowledge that our search is not exhaustive, since it is not a systematic review, we have found and reported beyond some significant examples of genes and SNPs in inflammatory loci potentially implicated in coronary disease.

## Genetic variants in inflammatory genes and coronary artery disease (CAD)

Regarding genes involved in inflammatory pathway, C-X-C motif ligand 12 (*CXCL12*), SH2B adaptor protein 3 (*SH2B3*), *AB0*, Human Leukocyte Antigen (*HLA*), interleukin 6 receptor (*IL-6R*), *IL-5*, Platelet endothelial cell adhesion molecule 1 (*PECAM1*), Protein C Receptor (*PROCR*) and antisense non-coding RNA in the INK4 locus (*ANRIL*) have been identified in different GWAS and sometimes confirmed in case-controls studies as significantly associated to coronary artery disease (CAD) (Table 1).

**Table 1:** Single nucleotide polymorphisms in inflammatory gene related to CAD.

**Locus**	**Gene(s)**	**Polymorphism(s) and association**	**Population(s)**	**References**
1	*IL6R*	rs4845625 (intron) *P* = 3.64 × 10^−10^	CARDIoGRAMplusC4D Consortium (*n* = 63,746 CAD patients; *n* = 130,681 HC)	CARDIoGRAMplusC4D Consortium et al., 2013
5q31.1	*IL-5*	rs2706399 (near gene) *P* = 2.1 × 10^−6^_(combined)_	IBC 50K CAD Consortium Discovery: (*n* = 15,596 European and South Asian CAD patients; *n* = 34,992 European and South Asian HC) Replication: (*n* = 17,121 CAD patients; *n* = 40,473 HC)	IBC 50K CAD Consortium, 2011
6p21.3	*HLA-C HLA-B HCG27*	rs3869109 (near gene) *P* = 1.12 × 10^−9^_(combined)_	Discovery: 5 European case control meta-analysis (OHGS_A, OHGS_CCGB_B, DUKE, WTCCC, ITH) (*n* = 7,123 CAD patients; *n* = 6,826 HC) Replication: 5 CAD European case control studies (GerMIFS1, GerMIFS2, PennCath, MedStar, OHGS_CCGB_S) (*n* = 5,211 CAD patients; *n* = 5,821 HC)	Davies et al., 2012
6p21.3	*HLA-C HLA-B HCG27*	rs3869109 (near gene) *P* < 0.05	Chinese Han population (*n* = 422 patients including 210 cases with coronary stenosis ≥50% or previous MI; *n* = 212 HC)	Xie et al., 2013
9p21.3	*ANRIL*	rs1333049 (near gene) 1.8 × 10^−14^	Wellcome Trust Case Control Consortium (WTCCC) (*n* = 1,926 CAD patients; *n* = 2,938 HC)	Burton et al., 2007
9p21.3	*ANRIL*	rs1333049 (near gene) *P* = 1.80 × 10^−14^ for WTCCC *P* = 3.40 × 10^−6^ for German study	WTCCC study (*n* = 1,988 MI patients or coronary revascularization < 66 years; *n* = 3,004 HC) German study (*n* = 875 MI patients < 60 years; *n* = 1,644 HC)	Samani et al., 2007
9p21	*ANRIL*	rs2383207 (intron variant) *P* = 2.0 × 10^−16^_(combined)_ rs10757278 (near gene) *P* = 1.2 × 10^−20^_(combined)_	Iceland A (*n* = 1,607 MI patients; *n* = 6,728 HC) Iceland B (*n* = 665 MI patients; *n* = 3,533 HC) Atlanta (*n* = 596 MI patients; *n* = 1,284 HC) Philadelphia (*n* = 582 MI patients; *n* = 504 HC) Durham (*n* = 1137 MI patients; *n* = 718 HC) All groups (*n* = 4,587 MI patients / *n* = 12,767)	Helgadottir et al., 2007
9p21	*ANRIL*	rs10757274 (near gene) *P* = 3.9 × 10^−6^_(combined)_ rs2383206 (intron variant) *P* = 3.9 × 10^−6^_(combined)_	Copenhagen City Heart Study (CCHS) (*n* = 1,525 CHD patients; *n* = 9,053 HC) Dallas Heart Study (DHS) (*n* = 154 CHD patients; *n* = 527 HC) Ottawa Heart Study population (OHS-3) (*n* = 647 CHD patients; *n* = 847 HC)	McPherson et al., 2007
9p21.3	*ANRIL*	rs10965215 (intron variant) *P* = 0.020 rs10738605 (nc transcript variant) *P* = 0.019	Chinese Han population (*n* = 286 MI patients; *n* = 646 HC)	Cheng et al., 2017
9q34.2	*AB0*	rs579459 (near gene) *P* = 4.08 × 10^−14^_(combined)_	14 GWAS Discovery: (*n* = 22,233 CAD patients; *n* = 64,762 European HC) Replication: (*n* = 30,949 CAD patients; *n* = 27,674 HC)	Schunkert et al., 2011
9q34.2	*AB0*	rs514659 (near gene) *P* = 7.62 × 10^−9^ and other 8 SNPs at genome wide significant level	*n* = 5,783 patients who had angiographic CAD and MI; *n* = 3,644 patients who had angiographic CAD but no MI	Reilly et al., 2011
9q34.2	*AB0*	rs579459 (near gene) *P* = 2.66 × 10^−8^	CARDIoGRAMplusC4D Consortium (*n* = 63,746 CAD patients; *n* = 130,681 HC)	CARDIoGRAMplusC4D Consortium et al., 2013
10q11.21	*CXCL12*	rs501120 (near gene) *P* = 1.31 × 10^−3^_(combined)_ rs1746048 (near gene) *P* = 2.96 × 10^−3^_(combined)_	WTCCC study (*n* = 1,926 MI patients or coronary revascularization < 66 years; *n* = 2,938 HC)	Burton et al., 2007
10q11.21	*CXCL12*	rs1746048 (near gene) *P* = 5.73 × 10^−7^_(combined)_ rs501120 (near gene) *P* = 9.46 × 10^−8^_(combined)_	WTCCC study (*n* = 1,988 MI patients or coronary revascularization < 66 years; *n* = 3,004 HC) German study (*n* = 875 MI patients < 60 years; *n* = 1,644 HC)	Samani et al., 2007
10q11.21	*CXCL12*	rs1746048 (near gene) *P* = 8.14 × 10^−11^_(combined)_	Discovery: MIGen (*n* = 2,967 early-onset MI cases (men ≤ 50 years and women ≤ 60 years); *n* = 3,075 HC) Replication: 6 other studies (*n* = 5,469 MI patients; *n* = 5,469 HC)	Kathiresan et al., 2009
10q11.21	*CXCL12*	rs501120 (near gene) *P* = 4.34 × 10^−4^_(combined)_	Coronary Artery Disease Consortium (*n* = 11,550 CAD patients; *n* = 11,205 HC)	Coronary Artery Disease Consortium et al., 2009
10q11.21	*CXCL12*	rs501120 (near gene) *P* = ns_(combined)_ Only women: *P* = 8.36 × 10^−3^	Chinese Han population (*n* = 2,335 CAD patients; *n* = 1,078 HC)	Xie et al., 2011
10q11.21	*CXCL12*	rs1065297 (3′ UTR) all *P* = 0.01;	Chinese Han population (*n* = 597 CAD patients; 685 HC)	Zhang et al., 2017
12q24	*SH2B3*	rs3184504 (Trp60Arg) *P* = 8.6 × 10^−8^	Six populations (*n* = 6,650 MI patients; *n* = 40,621 HC)	Gudbjartsson et al., 2009
12q24	*SH2B3*	rs3184504 (Trp60Arg) *P* = ns	FGENTCARD population (*n* = 1520 CAD patients; *n* = 424 HC)	Saade et al., 2011
12q24	*SH2B3*	rs3184504 (Trp60Arg) *P* = 5.44 × 10^−11^	CARDIoGRAMplusC4D Consortium (*n* = 63,746 CAD patients; *n* = 130,681 HC)	CARDIoGRAMplusC4D Consortium et al., 2013
12q24	*SH2B3*	rs653178 (intron variant) *P* = 2.2 × 10^−6^	CARDIoGRAM (*n* = 52,120 MI patients; *n* = 34,875 HC)	Olden et al., 2013
12q24	*SH2B3*	rs79105258 (near gene) *P* = 2.5 × 10^−5^	TAICHI Consortium (*n* = 3,133 Taiwan CAD patients; *n* = 5,423 HC)	Assimes et al., 2016
17	*PECAM1*	rs1867624 (near gene) *P* = 3.98 × 10^−8^	CARDIoGRAMplusC4D, EPIC-CVD, + 8 other studies and six studies from the Myocardial Infarction Genetics Consortium (MIGen) (*n* = 88,192 CAD patients; *n* = 162,544 HC)	Howson et al., 2017
20	*PROCR*	rs867186 (Ser219Gly) *P* = 2.70 × 10^−9^	CARDIoGRAMplusC4D, EPIC-CVD, + 8 other studies and six studies from the Myocardial Infarction Genetics Consortium (MIGen) (*n* = 88,192 CAD patients; *n* = 162,544 HC)	Howson et al., 2017

*ANRIL, antisense non-coding RNA in the INK4 locus; CAD, coronary artery disease; CXCL12, C-X-C motif ligand 12; HC, healthy controls; HLA, Human Leukocyte Antigen; IL-5, interleukin 5; IL-6R, interleukin 6 receptor; MI, myocardial infarction; PECAM1, Platelet endothelial cell adhesion molecule 1; PROCR, Protein C Receptor; SH2B3, SH2B adaptor protein 3*.

### *CXCL12* polymorphisms

*CXCL12* is a gene located on 10q11.1 encoding for a member of the alpha chemokine protein family, also called stromal cell-derived factor-1 (SDF-1). It is involved in vascular repair and remodeling through endothelial progenitor cell recruitment, and it is expressed in atherosclerotic lesion by contributing to macrophages migration that promote formation of complicated and unstable plaques by maintaining a pro-inflammatory microenvironment (Farouk et al., 2010).

The association between two SNPs (rs501120 and rs1746048) on 10q11.21 and CAD has been originally shown in the GWAS performed in the Wellcome Trust Case Control Consortium Study (WTCCC) involving 1,926 CAD patients and 2,938 controls (Burton et al., 2007) but not emphasized. In fact, both rs501120 [per allele odds ratio (OR) 1.24] and rs1746048 (OR 1.21) resulted associated with CAD but not at a genome wide significant level (Burton et al., 2007). In the same year, data of the Wellcome Trust Case Control Consortium and of the German Myocardial Infarction Family Study (enrolling 875 MI patients lower than 60 years and 1,644 healthy controls) were combined and confirmed previously published results (Samani et al., 2007) about these SNPs.

Kathiresan et al. published the results of a GWAS conducted in 2,967 early-onset MI cases (men ≤ 50 years and women ≤ 60 years) and in 3,075 age- and sex-matched controls enrolled in the Myocardial Infarction Genetics Consortium (MIGen), other than in an additional 6 studies comprising in total 5,469 MI cases and 5,469 controls (Kathiresan et al., 2009). The association between rs1746048 of the *CXCL12* gene and MI resulted highly significant (combined *p*-value 8.14 × 10^−11^).

By analyzing 11,550 cases and 11,205 controls from 9 European studies, the Coronary Artery Disease Consortium reported for the SNP rs501120 a nominally significant association (*P* = 4.34 × 10^−4^) and observed a possible sex interaction with a significant effect in women (OR = 1.29, 95% CI: 1.15–1.45, *P* = 1.86 × 10^−5^) but not in men (OR = 1.03, 95% CI: 0.96–1.11, *P* = 0.387; Coronary Artery Disease Consortium et al., 2009).

Successively, this observation has been replicated in a Chinese Han population of 2,335 coronary atherosclerosis patients and 1,078 controls undergoing coronary angiography. Accordingly, rs501120 at 10q11.21 was associated with coronary atherosclerosis in females (*P* = 8.36 × 10^−3^) but not in males (Xie et al., 2011).

In the same year, it was shown that SNPs rs1746048 and rs501120 of the *CXCL12* gene, are associated with high CXCL12 mRNAs and high CXCL12 plasma levels (Mehta et al., 2011) suggesting their functionality.

Very recently, in a case-control study involving 597 Chinese Han CAD patients and 685 healthy control, Zhang et al., by genotyping six SNPs (rs1065297, rs1801157, rs266089, rs197452, rs2839693, and rs10793538), identified three different SNPs (rs1065297, rs266089, and rs10793538) of *CXCL12* associated with the risk of CAD (Zhang et al., 2017). Stratified according to gender, in the allele model, rs266089 and rs2839693 in *CXCL12* gene were associated with the risk of CAD in men, while rs1065297 and rs10793538 in *CXCL12* gene were associated with the risk of CAD in women. However, after adjustment by sex and age this association resulted not yet significant.

Anyhow, although this gene appears to be quite interesting, most of the tested SNPs are not functional, and the conflicting results obtained prompt to be cautious about a solid implication of this gene in coronary atherosclerosis.

### *SH2B3* polymorphisms

*SH2B3*, also known as *LNK*, encodes for an intracellular adaptor protein expressed in vascular endothelial cells and functions as a negative regulator in many pathways, as cytokine signaling pathways (Fitau et al., 2006; Gueller et al., 2011). In a mouse model of atherosclerosis, it has been demonstrated that this gene is up-regulated over five-fold as endothelial cells change from normal to the atherosclerotic disease state (Erbilgin et al., 2013).

Gudbjartsson et al., by investigating sequence variants affecting eosinophil counts in blood of 9,392 Icelanders and in six different populations (6,650 cases and 40,621 controls) found that the SNP rs3184504 (R262W) in exon 3 of *SH2B3* located at 12q24, resulted significantly associated with MI (Gudbjartsson et al., 2009). Since eosinophils are leukocytes involved in initiation and propagation of inflammatory responses, the accumulation of eosinophils in the thrombus could contribute to the genesis and progression of thrombus (Jiang et al., 2015).

Saade et al. performed a replication study of nine previously identified CAD/MI susceptibility loci in a total of 2,002 subjects of the FGENTCARD population (Saade et al., 2011). Previously reported results about rs3184504 and rs653178 were not confirmed in this population.

The SNP rs653178 near *SH2B3* showed, in a very large population including participants from the CARDIoGRAM (*n* = 86,995) and the ICBP (*n* = 69,395), a significant association with CAD (*P* = 2.2 × 10^−6^; Olden et al., 2013).

With the aim to investigate the genetic susceptibility to CAD, in a large study (63,746 CAD cases and 130,681 controls) performed by combining genome-wide data from the CARDIoGRAM and C4D consortia, Deloukas and colleagues found evidence that only the SNP rs3184504 at the *SH2B3* locus displayed statistically significant association with CAD (CARDIoGRAMplusC4D Consortium et al., 2013).

Different results were more recently reported by Assimes et al. by genotyping 5,423 HC and 3,133 CAD patients from Taiwan (Assimes et al., 2016). Despite the association of rs79105258 (*P* = 2.5 × 10^−5^), it was observed that the rs79105258 of the *SH2B3* contributes to CAD only in the presence of the rs2289800 of *COL4A1*.

Ji et al. conducted a study on a Chinese Han population of 456 CAD patients (291 men, 165 women) and 685 age-matched controls (385 men, 300 women) to determine the influence of sex on the association between *SH2B3* polymorphisms and CAD. Despite *SH2B3* polymorphisms resulted associated with CAD susceptibility in both women and men (Ji et al., 2017), the authors observed that the *SH2B3* haplotypes were associated with decreased CAD risk in women (*P* = 0.007) but increased CAD risk in men (*P* = 0.047). Interestingly enough, the rs3184504 at the *SH2B3* locus is one of the 29 SNPs identified in the first consortium able to discover genetic loci consistently associated with BP/hypertension at a genome significant level depicting a possible link between hypertension and atherosclerosis (Ehret et al., 2011).

Also for this gene the first findings were not constantly replicated but, differently from *CXCL12*, at least the rs3184504 determines an amino acid change and is located inside the gene. Even if more research is needed, it sounds also attracting the common positivity in GWAS for hypertension.

### HLA polymorphisms

HLA is a set of cell surface proteins essential for the acquired immune system by playing an important role in inflammation and T cell responses and mediating the interactions between leucocytes.

An association between a *HLA* locus, 6p21.3, and CHD was demonstrated for the first time by a meta-analysis of 5 GWAS including 13,949 subjects of European ethnicity (7,123 cases, 6,826 control subjects). The SNP significantly associated with CAD, the rs3869109, was located in an intragenic region between *HLA-C* and *HCG27* (HLA complex group 27; Davies et al., 2012).

To investigate whether this SNP confers the risk of premature CAD in the Chinese Han population, this association was explored in a total of 422 patients including 210 cases with coronary stenosis ≥50% or previous MI (male < 55 years and female < 65 years) and 212 controls without documented CAD (Xie et al., 2013). The authors showed that rs3869109 G-carriers have a higher risk of premature CAD than AA homozygotes (OR: 1.997, 95% CI: 1.166–3.419, *P* = 0.012; OR 1.695, 95% CI: 1.044–2.752, *P* = 0.033; respectively) and that there was a significant association between rs3869109 genotypes and the severity of disease.

Sinisalo et al. by conducting a large-scale genetic analysis on a case-control cohort comprising 5,376 ACS cases and 4,852 unrelated controls from 4 populations of 2 European countries, found a risk haplotype for the disease containing single nucleotide polymorphisms from BTNL2 and HLA-DRA genes and the HLA-DRB1^*^01 allele (Sinisalo et al., 2016).

Taking together, the results of these studies are not convincing about a pivotal role of HLA in atherosclerosis.

### ABO

Among the 13 loci newly associated with CAD (P < 5 × 10^−8^) in the very large meta-analysis performed by Schunkert et al. in 14 GWAS comprising 22,233 CAD patients and 64,762 controls of European descent followed by genotyping of 56,682 additional individuals (Schunkert et al., 2011), the risk allele on chromosome 9q34.2 (rs579459) in the *ABO* gene resulted associated with CAD (*P* = 4.08 × 10^−14^) and weakly with increased LDL (*P* = 0.0049) and total cholesterol (*P* = 0.0038).

To identify loci that predispose to MI, Reilly et al. compared in a GWAS 5,783 patients who had angiographic CAD and MI and 3,644 patients who had angiographic CAD but no MI. They identified a novel association at the *ABO* locus: 11 *ABO* SNPs resulted significantly related to MI risk (Reilly et al., 2011). The authors hypothesized that the AB0 association could be attributable to the glycotransferase-deficient enzyme that encodes the AB0 blood group 0 phenotype previously proposed to protect against MI (Reilly et al., 2011). It has been hypothesized that blood group antigens could be directly involved in atherosclerotic inflammatory process (Wu et al., 2007). The same considerations as for the HLA locus apply also to the ABO locus.

### Other “inflammatory” loci associated with CAD

In 2007 the locus 9p21 was identified as the strongest genetic susceptibility locus for CAD and MI independently by three different research groups (Burton et al., 2007; Helgadottir et al., 2007; McPherson et al., 2007; Samani et al., 2007). One year later, Schunkert confirmed the association between chromosome 9p21.3 and CAD in 7 case-control studies involving 4,645 patients and 5,177 healthy controls (Schunkert et al., 2008).

The variants identified in these studies are located in a locus that contains several genes, like *CDKN2A* [coding for p16(ink4a) and p14(ARF)], *CDKN2B* [coding for p15(ink4b)], *MTAP* (Holdt et al., 2011). These proteins are cyclin-dependent kinase inhibitors involved in the regulation of cell proliferation, aging, senescence and apoptosis in many cell types and result abundantly expressed in atherosclerotic lesions (Holdt et al., 2011). In this locus also the gene antisense non-coding RNA in the INK4 locus (*ANRIL*) maps also called the CDKN2B antisense RNA 1.

Several SNPs in the 9p21.3 locus have been showed associated with CAD risk (Burton et al., 2007; Samani et al., 2007; Schunkert et al., 2011).

More recently, a consistent association between two variants in the 9p21.3 locus (rs10965215 and rs10738605) and CAD has been reported also in the Chinese Han population (Cheng et al., 2017). For rs10965215, unconditional logistic regression analysis revealed that the G allele increased MI risk with OR of 1.37 (95% CI = 1.05–1.78, *P* = 0.020). For rs10738605, C allele conferred increased MI risk with OR of 1.38 (95% CI = 1.06–1.80, *P* = 0.019) as compared to the G allele after adjustment for conventional risk factors (Cheng et al., 2017).

*ANRIL* is expressed in endothelial cells, smooth muscle cells, and inflammatory cells and is involved in the regulation of expression of adjacent protein coding genes, including *MTAP, CDKN2A* (p15INK4b), and *CDKN2B* (p16INK4a) through multiple mechanisms, including RNA interference, gene silencing, chromatin remodeling, or DNA methylation (Holdt et al., 2010).

This locus remains still one of the most promising for coronary atherosclerosis, being detected in most of the GWAS performed and subtending genes of putative effect. Nevertheless, the exact mechanism and the gene responsible is to be definitely proved.

The IBC 50K CAD Consortium examined 49,094 genetic variants in 2,100 genes in 15,596 CAD cases and 34,992 controls and replicate putative novel associations in an additional 17,121 CAD cases and 40,473 controls (IBC 50K CAD Consortium, 2011). Among the new variants identified, SNP (rs2706399) in interleukin 5 (*IL5*) gene (5q31.1) resulted statistically associated with CAD in the discovery and replication study (combined *p*-value 2.1 × 10^−6^). IL5 is an interleukin produced by T helper-2 cells with a role in the inflammatory process characterizing the development and progression of atherosclerosis and CAD (Hansson, 2005).

The association between rs4845625 of the *IL6R* and CAD (*P* = 3.64 × 10^−10^) was demonstrated in the CARDIoGRAMplusC4D Consortium, in which the CARDIoGRAM population (22,233 cases and 64,762 controls) was expanded with 34 additional European or south Asian population comprising 41,513 cases and 65,919 controls (CARDIoGRAMplusC4D Consortium et al., 2013). The IL6-mediated activation of IL6R, a receptor located on the leukocytes and hepatocytes membrane, stimulates the proinflammatory cascade including hepatic production of acute phase reactants C-reactive protein and fibrinogen (Schuett et al., 2009).

Moreover, the Asp358Ala (rs2228145) in IL6R was not associated with several traditional risk factors such as blood pressure, adiposity, hyperglycaemia, cholesterol, and smoking but with the concentration IL-6, C-reactive protein, fibrinogen, and coronary heart disease implying a causal role for this inflammatory pathway (IL6R Genetics Consortium Emerging Risk Factors Collaboration et al., 2012).

Howson et al. genotyped 56,309 participants and performed meta-analysis of results with 194,427 participants previously genotyped, totalising 88,192 CAD cases and 162,544 controls. The authors identified 25 new SNP–CAD associations (P < 5 × 10^−8^) from 15 genomic regions, including SNPs in *PECAM1* (rs1867624, *P* = 3.98 × 10^−8^) and in *PROCR* (rs867186, *P* = 2.70 × 10^−9^) genes, that are involved in cellular adhesion, leukocyte migration and inflammation (Howson et al., 2017).

In particular PECAM-1 is important to maintain the integrity of the vascular barrier. Accordingly, the consequence of a not conserved barrier can be the development of chronic inflammatory diseases such as atherosclerosis (Privratsky et al., 2011).

These are a few examples of how genetics can and will be able to in the near future identify molecules or pathways as tailored anti-inflammatory therapeutic targets in atherosclerosis.

## Conclusion

Despite the inflammatory core of atherosclerosis has been well described, a straightforward anti-inflammatory therapy for targeting inflammation is still missing in the physician's armamentarium. Therapy with biological drugs is attractive but probably too expensive to be largely available. Newer targets are needed and genetics can help with this issue. So far, the results coming from GWAS have pinpointed a few loci closed to inflammatory molecules and/or pathways consistently associated with atherosclerosis and CV events, revamping the strict link between inflammation and atherosclerosis suggesting some tailored target therapy.

In some cases Mendelian randomization studies can help in detecting which of them is a real primary therapeutic goal and which should be considered secondary (Ridker and Lüscher, 2014).

In the upcoming genomic era, both whole-exome and whole-genome sequencing will pinpoint new and old loci associated with atherosclerosis identifying new molecular targets or characterizing which inflammatory pathway could be a suitable target to block atherosclerosis even in its early stages.

## Author contributions

Both authors contributed to the drafting and completion of the final version of the manuscript.

### Conflict of interest statement

The authors declare that the research was conducted in the absence of any commercial or financial relationships that could be construed as a potential conflict of interest.
